# Ca^2+^-dependent calmodulin binding to cardiac ryanodine receptor (RyR2) calmodulin-binding domains

**DOI:** 10.1042/BCJ20180545

**Published:** 2019-01-18

**Authors:** Malene Brohus, Mads T. Søndergaard, Sui Rong Wayne Chen, Filip vanPetegem, Michael T. Overgaard

**Affiliations:** 1 Department of Chemistry and Bioscience, Aalborg University, 9220 Aalborg, Denmark; 2 Libin Cardiovascular Institute of Alberta, Department of Physiology and Pharmacology, University of Calgary, Calgary, Alberta T2N 1N4, Canada; 3 Department of Biochemistry and Molecular Biology, University of British Columbia, Vancouver, British Columbia V6T 1Z3, Canada

**Keywords:** calmodulin, intracellular calcium, ryanodine receptors

## Abstract

The Ca^2+^ sensor calmodulin (CaM) regulates cardiac ryanodine receptor (RyR2)-mediated Ca^2+^ release from the sarcoplasmic reticulum. CaM inhibits RyR2 in a Ca^2+^-dependent manner and aberrant CaM-dependent inhibition results in life-threatening cardiac arrhythmias. However, the molecular details of the CaM–RyR2 interaction remain unclear. Four CaM-binding domains (CaMBD1a, -1b, -2, and -3) in RyR2 have been proposed. Here, we investigated the Ca^2+^-dependent interactions between CaM and these CaMBDs by monitoring changes in the fluorescence anisotropy of carboxytetramethylrhodamine (TAMRA)-labeled CaMBD peptides during titration with CaM at a wide range of Ca^2+^ concentrations. We showed that CaM bound to all four CaMBDs with affinities that increased with Ca^2+^ concentration. CaM bound to CaMBD2 and -3 with high affinities across all Ca^2+^ concentrations tested, but bound to CaMBD1a and -1b only at Ca^2+^ concentrations above 0.2 µM. Binding experiments using individual CaM domains revealed that the CaM C-domain preferentially bound to CaMBD2, and the N-domain to CaMBD3. Moreover, the Ca^2+^ affinity of the CaM C-domain in complex with CaMBD2 or -3 was so high that these complexes are essentially Ca^2+^ saturated under resting Ca^2+^ conditions. Conversely, the N-domain senses Ca^2+^ exactly in the transition from resting to activating Ca^2+^ when complexed to either CaMBD2 or -3. Altogether, our results support a binding model where the CaM C-domain is anchored to RyR2 CaMBD2 and saturated with Ca^2+^ during Ca^2+^ oscillations, while the CaM N-domain functions as a dynamic Ca^2+^ sensor that can bridge noncontiguous regions of RyR2 or clamp down onto CaMBD2.

## Introduction

Cardiac ryanodine receptors (RyR2s) are large intracellular Ca^2+^ channels that control the release of Ca^2+^ from the sarcoplasmic reticulum (SR) in cardiomyocytes [1]. Excitation of cardiomyocytes causes a small influx of extracellular Ca^2+^ through voltage-gated Ca^2+^ channels (Ca_V_1.2s). This initial rise in cytosolic Ca^2+^ activates RyR2 and results in a Ca^2+^-induced Ca^2+^ release (CICR) sufficient to cause myofilament contraction [1,2]. Equally important, RyR2 inactivation allows for replenishing of the SR Ca^2+^ store in preparation for the next cardiomyocyte excitation [1–3]. RyR2 forms homotetrameric channels that arrange in spatially well-defined Ca^2+^ release units [1]. This organization means that the concentration of free Ca^2+^ in the cytosol near RyR2 oscillates between ∼0.2 µM at diastole and 200–400 µM during CICR and systole [2,4,5]. The cytosolic Ca^2+^-sensing protein calmodulin (CaM) binds to RyR2 and inhibits the release of Ca^2+^ in a Ca^2+^-dependent manner [1,6–10]. CaM consists of two Ca^2+^-binding domains (the N- and C-domains) that each contains two EF-hands, and thereby enables the binding of four Ca^2+^ ions. The two CaM domains display distinct Ca^2+^ affinities and -kinetics, and are connected by a flexible linker region that allows them to make independent interactions [10–14].

**Figure 1.**
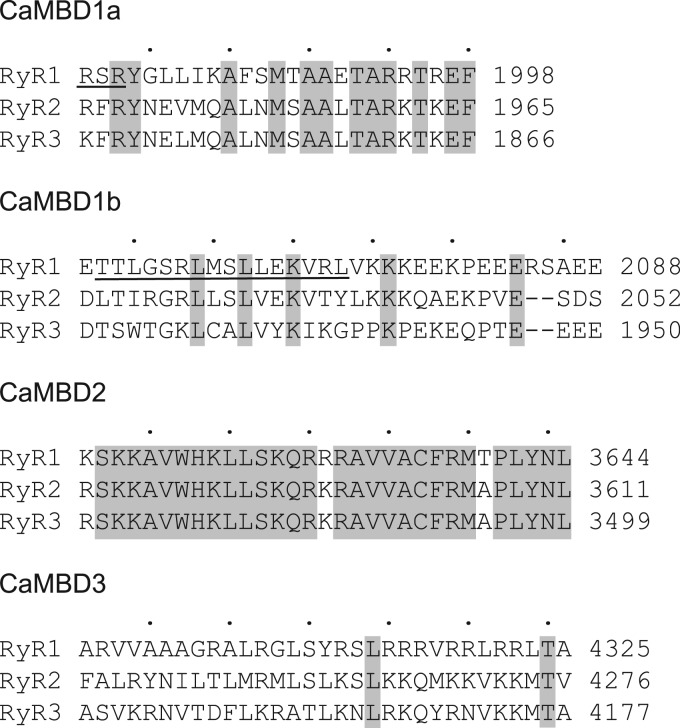
Overview of CaMBDs of RyR. ClustalW protein sequence alignment of the three human RyR isoforms (hRyR1–3). Amino acids that are identical between hRyR1-3 are highlighted in gray and amino acids that are resolved in the cryo-EM structure of rabbit RyR1 (PDB: 3J8H) are underlined. The dots above the alignment indicate amino acid intervals of five according to the RyR2 sequence.

Importantly, the skeletal muscle ryanodine receptor (RyR1) and RyR2 are differentially regulated by CaM. Whereas both channels display Ca^2+^-dependent regulation and are inhibited by CaM at higher Ca^2+^ levels, RyR1 is activated by CaM at low Ca^2+^ levels, while RyR2 is inhibited [6,15–17]. One CaM-binding domain (CaMBD) has been demonstrated to be central for the interaction between CaM and both RyR1 and RyR2, and also for the inhibition by CaM [6,8,10,15–18]. However, this CaMBD (RyR2 Arg-3581–Pro-3607, termed CaMBD2 in this study) is highly conserved between RyR isoforms (Figure 1) and is therefore unlikely to constitute the site of differential regulation by CaM. As such, the site(s) responsible for the different effects of CaM on the two RyR channels remain(s) unresolved.

Even though the molecular details of the interaction between RyR2 and CaM are not fully understood, it has been demonstrated that Ca^2+^ saturation of both CaM domains is required for full inhibitory effect [8,10,15,18–21]. Moreover, the Ca^2+^ affinities of each CaM domain in the presence of RyR2 CaMBD2 differ by ∼30-fold, with the C-domain having the highest Ca^2+^ affinity, and implies distinct roles of each domain in the regulation of RyR2 [10]. Experiments examining the binding of CaM to RyR2 fragments support up to four CaMBDs, here referred to as CaMBD1a, -1b, -2, and -3 (Figure 1 and Table 1) [9,22–31]. Recently, Lau et al. [28] analyzed the interaction between CaM and RyR2 CaMBD1a, -2, and -3 by isothermal titration calorimetry (ITC). The measurements were carried out at Ca^2+^-free [10 mM ethylenediaminetetraacetic acid (EDTA)] and Ca^2+^-saturating (10 mM CaCl_2_) conditions, and all CaMBDs were fused to maltose-binding protein (MBP). CaMBD1b has so far not been investigated beyond CaM overlay assays [22].

**Table 1:** RyR2 CaMBD peptides used in the present study CaMBD2(+) peptides were used for determining the importance of CaMBD2 hydrophobic anchors. Numbering corresponds to human RyR2 (UniprotKB Q92736).

Nomenclature	RyR2 residues	Peptide sequence (length)	Mutation/modification	Ref.
CaMBD1a	F1942 – R1966	RFRYNEVMQALNMSAALTARKTKEF (25)		[28,34]
CaMBD1b	D2022 – S2052	DLTIRGRLLSLVEKVTYLKKKQAEKPVESDS (31)		[22,46]
CaMBD1b + P	D2022 – S2052	DLTIRGRLL**S**LVEKVTYLKKKQAEKPVESDS (31)	S2031 phosphorylated	[22,46]
CaMBD2	R3581 – P3607	RSKKAVWHKLLSKQRKRAVVACFRMAP (27)		[28,44]
CaMBD2(+)	R3581 – L3611	RSKKAVWHKLLSKQRKRAVVACFRMAPLYNL (31)		[10,21,35]
CaMBD2(+)-W/A	R3581 – L3611	RSKKAV**A**HKLLSKQRKRAVVACFRMAPLYNL (31)	W3587A	
CaMBD2(+)-F/A	R3581 – L3611	RSKKAVWHKLLSKQRKRAVVAC**A**RMAPLYNL (31)	F3603A	
CaMBD3	F4246 – V4276	FALRYNILTLMRMLSLKSLKKQMKKVKKMTV (31)		[28]

In this study, we extended the investigation of the interaction between CaM and the four proposed RyR2 CaMBD peptides using a fluorescence anisotropy-based titration method. This method allowed us to explore the Ca^2+^ dependency of the interaction between CaM and RyR2 CaMBDs across a broad range of free Ca^2+^ concentrations ([Ca^2+^]), including the physiologically relevant range for the cardiomyocyte cytosol. Our results showed that the strength of each CaM–CaMBD interaction is highly Ca^2+^-dependent with higher [Ca^2+^] consistently resulting in higher affinity of CaM for the CaMBDs. Also, the interaction affinities and their Ca^2+^ dependencies varied significantly between the different CaMBDs, with CaM displaying the highest affinity for CaMBD2 and -3. Measurements using isolated CaM domains established that the CaM C-domain preferentially binds to CaMBD2 and the N-domain to CaMBD3. Finally, the CaM affinity for Ca^2+^ was markedly increased in the presence of the CaMBDs, compared with free CaM, and differed markedly between the two CaM domains.

## Experimental procedures

### Visualization of CaMBDs in the RyR structure and protein sequence alignment

The crystal structure of Ca^2+^–CaM in a complex with RyR1 CaMBD2 (PDB: 2BCX) was juxtaposed with the high-resolution cryo-EM structure of the closed state of rabbit RyR1 (rRyR1, PDB: 3J8H) [29,32]. The indicated position and orientation of CaM is according to the existing literature [23–27]. For protein sequence alignments and comparisons, rRyR1 and human RyR1–3 (hRyR1–3) were aligned using the ClustalW algorithm within the CLC Main Workbench 6 (CLC Bio, version 6.9.1) [33]. UniprotKB accession numbers: P11716, P21817, Q92736, and Q15413. The alignment of rRyR1 and hRyR2 was used to identify the hRyR2 amino acids equivalent to those in the rRyR1 cryo-EM structure and the alignment of hRyR1–3 was used to determine the conservation of CaMBDs between hRyRs. Delimitation of the protein regions corresponding to CaMBD1a, CaMBD1b, CaMBD2, and CaMBD3 were adopted from previous studies [15,22,28,34].

### Protein expression and purification

Expression and purification of full-length CaM and CaM domains was done as previously described and summarized below [28,35].

Full-length CaM (CaM: A2-K149) was expressed in *Escherichia coli (E. coli)* Rosetta (DE3) from a modified pMal vector with an N-terminal MBP and a tobacco etch virus (TEV) cleavage site. The MBP–TEV–CaM fusion protein was purified on an amylose affinity column (New England Biolabs) and subsequently cleaved by a TEV protease. Following cleavage, CaM, TEV, and MBP were separated by anion ion exchange chromatography using a Q-sepharose column (GE Healthcare). The CaM-containing fractions were pooled and concentrated before removing residual Ca^2+^ by adding 20 mM EDTA to the sample prior to applying it to a Superdex 75 size exclusion column (GE Healthcare).

CaM domains (CaM N-domain: SNAWGG-M1-R75; CaM C-domain: SNA-K76-K149) were expressed in *E. coli* Rosetta (DE3) from a modified pET28 vector with an N-terminal His-tag and a TEV cleavage site. The His–TEV–CaM protein was first purified by immobilized metal affinity chromatography (IMAC) using a Poros20 MC column (Applied Biosystems) and subsequently cleaved with a His-tagged TEV protease. The His-tagged TEV protease and cleavage product were removed in a second IMAC step and the flow-through, containing CaM domain, was further purified on a phenyl-sepharose and a Q-sepharose column (GE Healthcare). Lastly, the CaM domain pool was concentrated and applied to a Superdex 200 size exclusion column (GE Healthcare) for a final purification and Ca^2+^ removal step. The identity, purity, and integrity of each protein preparation were confirmed by SDS–PAGE (sodium dodecyl sulfate–polyacrylamide gel electrophoresis) and MALDI-TOF (matrix-assisted laser desorption/ionization-time-of-flight) mass spectrometry of trypsin-digested proteins and entire nondigested domains. Protein concentrations were measured using absorption at 280 nm (extinction coefficient 2560 cm^−1^ M^−1^ for intact CaM and the CaM C-domain; 5690 cm^−1^ M^−1^ for the N-domain, due to the added Trp residue).

### Peptides corresponding to RyR2 CaMBDs

Peptides with an N-terminal 5-TAMRA (5-carboxytetramethylrhodamine) label were from Proteogenix (>95% purity, Table 1). Peptide concentrations were determined from the 5-TAMRA absorption at 556 nm (extinction coefficient 103 000 cm^−1^ M^−1^). Stock solutions were kept in 5% acetonitrile and 0.1% trifluoroacetic acid. For investigating CaMBD2 hydrophobic anchor residue effects, an elongated version of CaMBD2 (CaMBD2(+)) was used along with peptide variants where the Trp or Phe residues were replaced with Ala (Table 1).

### pH- and Ca^2+^-buffered solutions

pH- and Ca^2+^-buffered solutions (pCa buffers) contained 50 mM HEPES [4-(2-hydroxyethyl)-1-piperazineethanesulfonic acid], 100 mM KCl, 0.5 mM EGTA [ethylene glycol-bis(β-aminoethyl ether)-*N*,*N*,*N*′,*N*′-tetraacetic acid], and 2 mM NTA (nitrilotriacetic acid) at pH 7.2 (25°C) and variable concentrations of CaCl_2_. Before dilution and pH adjustment, the batch of buffer was split and one aliquot was added CaCl_2_ to 3 mM total Ca^2+^ ([Ca^2+^]_tot_). Mixing of various amounts of the pCa buffers with and without 3 mM CaCl_2_ to different [Ca^2+^]_tot_ established various EGTA/NTA-buffered free Ca^2+^ concentrations ([Ca^2+^]) [36]. In practice, 1.5× concentrated pCa buffer stocks were prepared, and CaMBD and reducing agent (0.3 mM DTT) were added to the double distilled water used for diluting these concentrated buffers. The calculated buffer ionic strength was 150 mM, and the [Ca^2+^] was verified by Ca^2+^ titrations in the presence of CaM or CaM/RyR2–CaMBD2 [10].

### Titrations of CaMBD peptides with CaM, or CaM domains, at discrete Ca^2+^ concentrations

A two-dimensional titration assay was employed to determine the affinity of CaM, or isolated CaM domains, for binding to RyR2-derived CaMBD peptides at 16 discrete [Ca^2+^] [37]. This assay was based on measuring the change in the fluorescence anisotropy (FA) signal of the 5-TAMRA-labeled peptides occurring when CaM or CaM domain binds (Figure 3A.2). Measurements were done in 384-well microtiter plates (16 × 24 wells) with the CaMBD concentrations kept constant (∼50 nM), and varying the CaM concentration in 23 serial dilution steps across the columns of one plate row (Figure 3A.1). Titrations were done in pCa buffer (see above), which allowed for mixing high and low [Ca^2+^]_tot_ solutions to obtain specific [Ca^2+^], and each of the 16 rows in the microtiter plate was used for a defined [Ca^2+^] condition (Figure 3A.1–2). All in all, each microtiter plate allowed for 24 titrations points for CaM concentrations at each of the 16 different [Ca^2+^], i.e. 16 CaM–CaMBD titration curves (Figure 3B.1–2).

In practical detail, 3 µl of purified CaM (∼600–1200 µM) was manually aliquoted to each well in column 1 of a 384-well low-binding microtiter plate (Corning, Cat. #3575). Using an automated liquid handling robot (Hamilton, Microlab STARlet), 65 µl of the 16 different pCa buffers ([Ca^2+^] = 0.3 nM–400 µM), including 50 nM TAMRA-labeled CaMBD and 0.3 mM DTT, were aliquoted into each row in column 1, containing CaM. Next, 30 µl of each of the same 16 different pCa buffers were aliquoted into the 23 empty wells in each row. Finally, CaM in column 1 was serial diluted by row-wise transferring 38 µl volumes to the adjacent column, including 4 × 30 µl pre-mixing during aspiration. pCa buffer (38 µl) was discarded from the last column and produced 24 dilutions of CaM for each [Ca^2+^]. The titration procedure completed in <50 min and the protein dilution factor (38 : 68 µl) between columns was verified using fluorescein (Sigma–Aldrich, Cat. #F6377) in 10 mM NaOH. Immediately after mixing, the FA signal was measured in a fluorescence plate reader (Tecan, Infinite M1000) with excitation and emission wavelengths at 530 and 582 nm with 5 and 20 nm bandwidths, respectively. The signal gain was fixed at 95, the settling time was set to 200 ms, the FA *G*-factor to 1.144 (fluorescein determined), and 20 flashes were used per measurement. All combinations of CaM, isolated CaM domains, and CaMBDs were measured in at least duplicate. Plate mixing and measurements were done at 25°C.

### Titration curve analysis for determining the affinity of CaM or CaM domains for binding to CaMBD peptides

CaM and CaM domains were expected to bind RyR2 CaMBD peptides (P) stoichiometrically [28] with the binding affinity expressed as the apparent CaM/CaMBD complex (PCaM) dissociation constant (*K*_D_), i.e.

PCaM⇄P+CaM

$$K_{D}=\frac{\left[\text{P}]\cdot [\text{CaM}\right]}{\left[\text{PCaM}\right]}$$

where [P], [CaM], and [PCaM] are the concentrations of free CaMBD, free CaM, and CaM/CaMBD complex, respectively. This simple binding model assumes one type of CaM–CaMBD interaction, i.e. characterized by one *K*_D_. For this 1 : 1 interaction, the fractional saturation (*Y*) of CaMBD with CaM is given by the following equation:

1 $$Y=\frac{\left[\text{PCaM}\right]}{{\left[\text{P}\right]}_{\mathrm{tot}}}=\frac{K_{D}+{\left[\text{P}\right]}_{\mathrm{tot}}+{\left[\text{CaM}\right]}_{\mathrm{tot}}}{2\cdot {\left[\text{P}\right]}_{\mathrm{tot}}}-\sqrt{{\left(\frac{K_{D}+{\left[\text{P}\right]}_{\mathrm{tot}}+{\left[\text{CaM}\right]}_{\mathrm{tot}}}{2\cdot {\left[\text{P}\right]}_{\mathrm{tot}}}\right)}^{2}-\frac{{\left[\text{CaM}\right]}_{\mathrm{tot}}}{{\left[\text{P}\right]}_{\mathrm{tot}}}}$$

where [P]_tot_ and [CaM]_tot_ are the total concentrations of CaMBD peptide and CaM, respectively. For each plate row in the titration scheme described above, a titration curve of FA as a function of [CaM]_tot_ was obtained (Figure 3A.1–3). The measured FA signal consists of the FA signal from the free CaMBD peptide (FA_P_) and the FA signal from the CaM/CaMBD complex (FA_PCaM_), hence

2 $$\text{FA}=\text{F}{\text{A}}_{P}\cdot (1-Y)+\text{F}{\text{A}}_{\mathrm{PCaM}}\cdot Y$$

where FA_P_ and FA_PCaM_ represent the minimum (entirely free CaMBD peptide) and maximum (entirely bound CaMBD peptide) FA signal. Substituting the expression for *Y* in equation 1 for *Y* in equation 2 and isolating for FA allowed for fitting the 1 : 1 binding model to each titration curve and thus obtaining a value of *K*_D_ at that [Ca^2+^] (Figure 3A.3). *K*_D_ as a function of [Ca^2+^] was obtained by fitting each row-wise titration curve (Figure 3B.1–3) using nonlinear regression in GraphPad Prism 6.07. A potential interference of the 5-TAMRA label with the binding of CaM to the CaMBDs was excluded by a titration of free 5-TAMRA with CaM which showed no binding (data not shown).

CaM binding to RyR2 CaMBD1a did not fit well with a 1 : 1 interaction at [Ca^2+^] >800 nM, yet the observed maximum FA_PCaM_ values were highly similar to those for CaM bound to CaMBD1b, CaMBD2 (at low [Ca^2+^], see ‘Results’), and CaMBD3 peptides (FA range 240–260). By inferences, the binding of CaM to CaMBD1a was therefore likely an 1 : 1 interaction since the FA signal correlates with the protein–peptide geometric size [38]. The simplest model of 1 : 1 interaction assuming more than one type of CaM–CaMBD interaction has two different modes of CaM binding to CaMBD, each characterized by their own affinity and *K*_D_ values (*K*_DI_ and *K*_DII_). In this model, *Y* is defined as follows:

3 $$Y=\left(\frac{\left[\text{CaM}\right]}{K_{\mathrm{DI}}+[\text{CaM}]}+\frac{\left[\text{CaM}\right]}{K_{\mathrm{DII}}+[\text{CaM}]}\right)$$

[CaM] is not explicitly known in the titrations, but, for low-affinity interactions, [CaM] ≈ [CaM]_tot_ is a good approximation, and model fitting for CaMBD1a at [Ca^2+^] > 800 nM was done with this alternate expression for *Y* in equation 2. We considered only the first mode of CaM–CaMBD1a interaction with the highest affinity of the two (i.e. *K*_DI_), given that the *K*_DII_ values were 5–25-fold higher than *K*_DI_.

Differences in the fitted *K*_D_ values for CaM binding to the various CaMBD peptides were evaluated using the conservative measure of nonoverlapping 95% confidence intervals (Supplementary Tables S1 and S2). Anisotropy signals depend on the fluorescence emitter tumbling rate which, in turn, depends on the geometric size of the CaMBD peptide and CaM/CaMBD complex in solution [38]. However, in our experiments, the total fluorescence intensity of the 5-TAMRA probe was enhanced by Ca^2+^–CaM binding to CaMBD2 (data not shown). This property did not interfere with the measurement of *Y* via the FA signal, but did impede an accurate determination of the CaM/CaMBD2 complex size based on the FA_PCaM_ signal.

### Estimation of apparent Ca^2+^ affinities of CaM–peptide complexes

We determined the apparent affinities of CaM/CaMBD complexes for Ca^2+^ under conditions with an excess of CaM. In practice, FA signals as a function of [Ca^2+^] (column-wise curves) were extracted for a specific CaM : peptide ratio (∼4 for full-length CaM and ∼40 for isolated CaM domains):

4 $$\text{FA}=\text{F}{\text{A}}_{\mathrm{lowCa}}\cdot (1-Y)+\text{F}{\text{A}}_{\mathrm{highCa}}\cdot Y$$

where FA_lowCa_ and FA_highCa_ represent the FA signal at the lowest and highest [Ca^2+^], respectively, for each particular CaM concentration. These curves were fitted to a generic Hill model [39]:

5 $$Y=\frac{1}{{(\text{app}K_{D}/\left[\text{C}{\text{a}}^{2+}\right])}^{n}+1}$$

where *Y* is the fractional saturation of CaM/CaMBD complex with Ca^2+^, *n* the Hill coefficient, and app*K*_D_ is the [Ca^2+^] at half-saturation. Substituting the expression for *Y* in equation 4 for *Y* in equation 5 allowed for fitting the Hill model and thus obtaining an app*K*_D_ for Ca^2+^-binding to CaM/CaMBD complexes. Fitting was performed to the raw FA signals as a function of [Ca^2+^] using nonlinear regression in GraphPad Prism 6.07. The significance of the differences in the fitted app*K*_D_ for CaM and isolated CaM domains was evaluated using the conservative measure of nonoverlapping 95% confidence intervals (Supplementary Table S3). The fit was visualized using normalized fractional saturation (Figure 8).

## Results

### Visualization of CaM and CaMBDs in the three-dimensional structure of RyR

**Figure 2.**
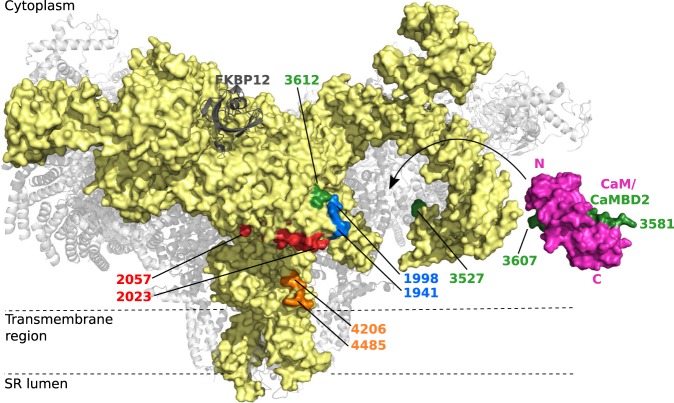
Approximate locations of CaMBDs in the rabbit RyR1 (rRyR1) structure. Ca^2+^-saturated CaM (purple) bound to RyR1 CaMBD2 (green) (PDB: 2BCX) was juxtaposed with the 3.8 Å resolution cryo-EM structure of the closed-state rabbit RyR1 homotetramer (PDB: 3J8H) [29,32]. One RyR1 monomer is shown in yellow surface representation, and the other three monomers as gray semi-transparent secondary structure schematics. The CaMBDs are not fully resolved, but the nearest resolved N- and C-terminal amino acids are marked by sequence position and color: CaMBD1a in blue, CaMBD1b in red, CaMBD2 in green, and CaMBD3 in orange. The labeled amino acid positions are numbered according to the equivalent human RyR2 positions.

The ability of CaM to interact with the four suggested CaMBDs was evaluated by comparing the locations of each CaMBD in the rabbit RyR1 (rRyR1) cryo-EM structure with the demonstrated CaM-binding site around CaMBD2 (Figure 2). The size of CaM relative to the RyR channel is indicated in Figure 2 by juxtaposing the channel and the crystal structure of Ca^2+^-saturated CaM in complex with the RyR1 CaMBD2 (CaM/RyR1CaMBD2). The suggested positioning of CaM as well as the orientation of its two domains are based on information from the available literature (Figure 2) [24–26,29,32]. Moreover, the orientation of CaMBD2 in the CaM/RyR1CaMBD2 complex was aligned with the positions of resolved amino acids most adjacent to CaMBD2 in the RyR1 structure.

Unfortunately, none of the four CaMBDs are fully resolved in the current RyR1 or RyR2 cryo-EM structures [29,30,40–43]. Instead, the amino acids closest to either end of each CaMBD were individually colored and labeled with numbers corresponding to the human RyR2 sequence (Figures 1 and 2 and Table 1). In this way, the general locations of each CaMBD could be reasonably inferred. For simplicity, RyR amino acid numbering corresponding to the human RyR2 sequence is used in this manuscript.

The three N-terminal amino acids of RyR2 CaMBD1a (Arg-1941, Phe-1942, and Arg-1943) are resolved and visible in the structure, but the adjacent RyR region from Tyr-1944 to Leu-1997 is unresolved (Figure 2, blue markings). CaMBD1b is largely resolved with Leu-2023 to Tyr-2038 visible in the RyR structure (Figure 2, red markings). The first N-terminal RyR2 CaMBD1b residue, Asp-2022, is part of the unresolved Glu-2013 to Asp-2022 region, and the C-terminal part of human RyR2 CaMBD1b (Leu-2039 to Ser-2052) is in the unresolved Leu-2039 to Ser-2056 region. Amino acids corresponding to CaMBD2 are all within the unresolved Gln-3527 to Leu-3611 region in the RyR structure. However, Pro-3612 is visible, indicating an approximate location for CaMBD2 (Figure 2, green markings). The location of CaMBD3 is poorly defined with all amino acids encompassed in the 278 amino acid long unresolved human RyR2 Ser-4207 to Ile-4484 region. The resolved human RyR2 amino acid closest to CaMBD3 is Ile-4206, which is 39 residues N-terminal to CaMBD3 Phe-4246 (Figure 2, orange markings).

Based on previous studies, CaM binds inside a pocket underneath the RyR helical domains (HD1–2) and between the central and handle domains (Figure 2) [29]. With this positioning, CaM is in close vicinity of RyR CaMBD1a, -1b, and -2, all located in, or close to, the handle domain. Additionally, RyR CaMBD2 appeared to span this CaM-binding pocket [44]. In comparison, the resolved residues closest to RyR CaMBD3 were further away from the CaM-binding pocket. However, assuming the 39 amino acids between human RyR2 Ile-4206 and Phe-4246 of CaMBD3 fold into a regular α-helix, this would span 59 Å (3.6 amino acids/turn, pitch of 5.4 Å) and thereby easily be able to extend to within the CaM-binding pocket (Figure 2).

In summary, investigating the approximate positions of the CaM-binding sites in the high-resolution RyR cryo-EM structure support that CaMBD1–3 are all within the range of the proposed CaM-binding pocket in RyR. Moreover, the lack of cryo-EM structure resolution, especially around CaMBD2 and -3, is consistent with these regions containing disordered, flexible CaMBDs likely to be accessible for CaM binding.

### CaM binds to RyR2 CaMBD2 and -3 with high affinities at physiological Ca^2+^ concentrations

**Figure 3.**
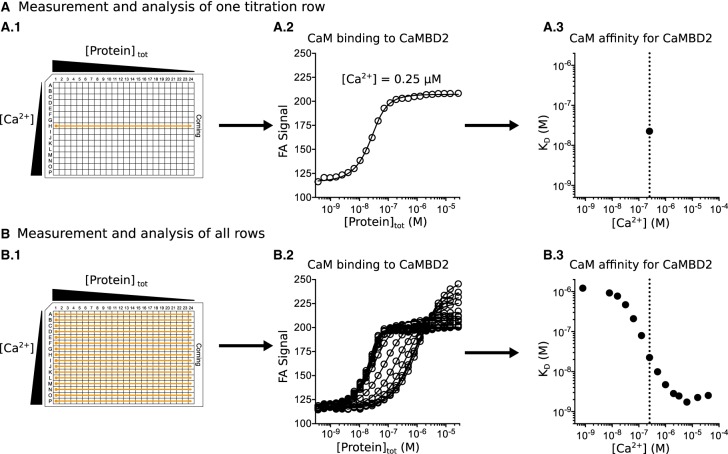
Measurement and analysis of titration curves for the determination of Ca^2+^-dependent dissociation constants (*K*_D_). (**A**) Simplified measurement and analysis example. Each row in the microtiter plate (**A.1**) contained a titration series of peptide with decreasing protein concentration (columns 1–24) at one [Ca^2+^]. Measuring the FA signal from each well in the row gave a titration curve, i.e. the FA signal as a function of the total protein concentration ([Protein]_tot_, **A.2**). The *K*_D_ for the protein binding to peptide at that particular [Ca^2+^] was obtained by fitting a stoichiometric binding model to the titration curve (**A.3**). (**B**) By repeating this fitting procedure for the titration curves from each row (**B.1–2**), the *K*_D_ as a function of [Ca^2+^] was obtained (**B.3**). Note that the plots in **A.3** and **B.3** are double-logarithmic.

**Figure 4.**
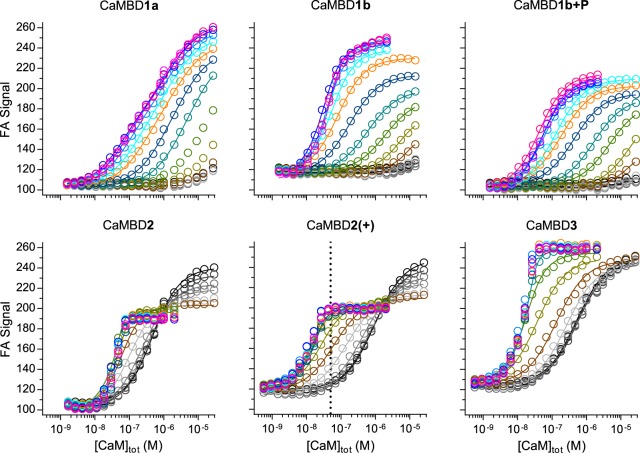
Example FA signal curves for the titration of RyR2 CaMBD peptides with CaM. The FA signal from the TAMRA-labeled peptide increased with CaM binding (symbols), and the titration curves were fitted to a stoichiometric binding model (solid lines)—see ‘Experimental Procedures’.

We titrated CaM in the presence of TAMRA-labeled CaMBD peptides at 16 discrete free Ca^2+^ concentrations ([Ca^2+^] = 0.3 nM–400 µM) (Figure 3 and Table 1). Binding of CaM to the CaMBD peptides was monitored as an increase in the FA of TAMRA. A protein–peptide binding model was fitted at each Ca^2+^ concentration to determine the apparent Ca^2+^-dependent affinities (*K*_D_) of the four CaM–CaMBD interactions (Figures 3 and 4, and Supplementary Table S1). Generally, this assay allowed us to reliably measure affinities from ∼1 nM to 5 µM.

**Figure 5.**
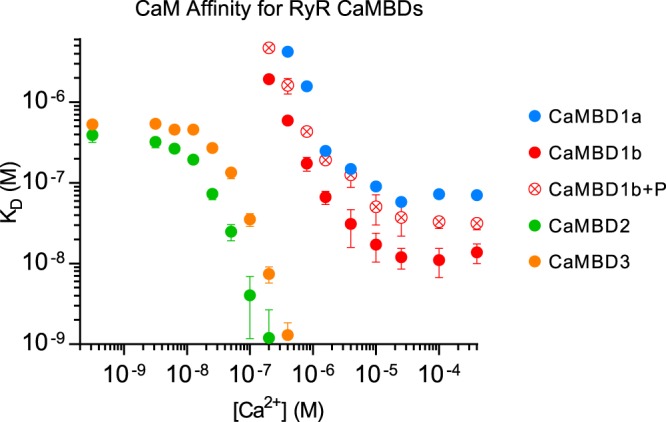
Ca^2+^-dependent affinities of CaM for binding to CaMBD1a, 1b, 2, and 3. The binding model-fitted affinities (*K*_D_) are plotted as a function of [Ca^2+^]. CaMBD color codings correspond to those indicated in Figure 2. Note the double logarithmic axes. Error bars represent the 95% confidence intervals of two (CaMBD1a and -1b ± P) or three (CaMBD2 and -3) replicates.

Binding curve analyses revealed that CaM bound to CaMBD2 and -3 with high affinities that increased with increasing [Ca^2+^] (Figure 5 and Supplementary Table S1). In fact, CaM affinities for CaMBD2 or -3 peptides were too high for reliable model fitting at [Ca^2+^] >0.2 µM and >0.4 µM, respectively (*K*_D_ < 1 nM). The CaM affinity for CaMBD2 increased 390-fold (*K*_D_ from 390 to 1 nM) when [Ca^2+^] was raised from 0.3 to 200 nM. Similarly, the CaM affinity for CaMBD3 increased 76-fold (*K*_D_ from 530 to 7 nM) in the same [Ca^2+^] range. Noteworthy, the CaM affinity for CaMBD2 was 2- to 9-fold higher than that for CaMBD3 in the low [Ca^2+^] range from 13 to 200 nM where a direct comparison was possible. This indicates that CaM preferentially binds to CaMBD2 at least in the low physiological [Ca^2+^] range.

### CaM binds to RyR2 CaMBD1a and -1b markedly weaker than to CaMBD2 and -3 at physiological Ca^2+^ concentrations

The CaM affinities for CaMBD1a or -1b were substantially lower than those for CaMBD2 or -3, with binding only detectable at ≥0.4 µM and ≥0.2 µM [Ca^2+^], respectively (Figure 5 and Supplementary Table S1). Nonetheless, the binding of CaM to both CaMBD1a and -1b was clearly Ca^2+^-dependent and affinities increased 34- and 42-fold, respectively, with the elevation of [Ca^2+^] from 0.4 to 400 µM. On average, the CaM affinity for CaMBD1b was 5-fold greater than that for CaMBD1a in the [Ca^2+^] range from 0.4 to 400 µM. Interestingly, phosphorylation of the RyR2 Ser-2031 present in CaMBD1b (CaMBD1b + P) lowered the affinity for CaM by ∼3-fold. Noteworthy, the CaM affinities for CaMBD1a, -1b, and -1b + P were approximately three orders of magnitude lower than those for CaMBD2 or -3 in the [Ca^2+^] range from 0.2 to 0.4 µM where a direct comparison was possible. These data demonstrate that out of the four CaMBDs investigated, CaM preferentially binds to CaMBD2 or -3 throughout the entire range of [Ca^2+^].

### CaM N- and C-domains do not contribute equally to the interactions with CaMBD2 or -3

**Figure 6.**
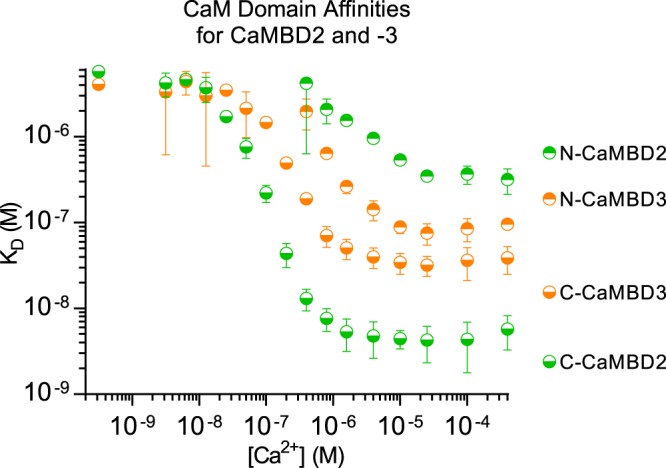
Ca^2+^-dependent affinities of CaM N- and C-domains for binding to CaMBD2 and -3. The binding model-fitted affinities (*K*_D_) are plotted as a function of [Ca^2+^]. CaMBD color codings correspond to those indicated in Figure 2. Note the double logarithmic axes. Error bars represent the 95% confidence intervals of two (CaMBD3) or three (CaMBD2) replicates.

Domain-wise contributions to the interaction between CaM and RyR2 CaMBD2 or -3 were investigated by determining the affinities of the isolated CaM N- or C-domain for either peptide (Figure 6 and Supplementary Table S2). Generally, each individual CaM domain bound to CaMBD2 and -3 with lower affinities than full-length CaM. Moreover, the CaM N-domain bound with much lower affinity than the C-domain, and binding could only be measured at [Ca^2+^] ≥0.4 µM.

The CaM C-domain displayed a Ca^2+^-dependent affinity (*K*_D_ from 5.7 µM to 4 nM) for CaMBD2 which was, on average, 27-fold lower than the full-length CaM affinity in the comparable range of [Ca^2+^]. Interestingly, the differences between the full-length CaM and C-domain affinities varied considerably across the measurable range of [Ca^2+^] (compare Supplementary Tables S1 and S2). At low nM [Ca^2+^], the full-length CaM affinity for CaMBD2 was ∼15-fold higher than for the isolated C-domain, whereas it was ∼50-fold higher at [Ca^2+^] ≥ 100 nM. This indicates that the binding of the Ca^2+^-free CaM C-domain to CaMBD2 is less dependent on the presence of the N-domain than the binding of the Ca^2+^-saturated CaM C-domain. The CaM N-domain bound to CaMBD2 with three orders of magnitude lower affinity than full-length CaM (at [Ca^2+^] from 0.8 to 1.6 µM, for CaMBD2 where comparison was possible).

Observations similar to those for CaMBD2 were made for CaMBD3. For the interaction between the isolated CaM C-domain and CaMBD3, the Ca^2+^-dependent affinities (*K*_D_ from 4 µM to ∼30 nM) were, on average, 36-fold lower than for full-length CaM, but ranging from ∼6-fold lower to ∼70-fold lower when [Ca^2+^] was raised from low nM to ≥100 nM. Also for CaMBD3, the isolated CaM N-domain exhibited a three orders of magnitude lower affinity for binding than full-length CaM (at a [Ca^2+^] of 0.4 µM where comparison was possible).

The observation that either isolated CaM domain had markedly lower affinity for binding to CaMBD2 and -3 than full-length CaM showed that both domains contributed to the binding of full-length CaM to either peptide. On the other hand, the contributions from each domain appeared far from equal. Thus, the CaM C-domain was the main contributor to the binding of intact CaM to both CaMBD2 and -3. In addition, the CaM C-domain bound to CaMBD2 with, on average, 9-fold higher affinity than to CaMBD3 in the [Ca^2+^] range from 0.1 to 400 µM. Hence, the isolated CaM C-domain showed a clear preference for CaMBD2 over CaMBD3 across the physiological [Ca^2+^]. Importantly, the opposite preference was observed for the CaM N-domain in the measurable [Ca^2+^] range from 0.4 to 400 µM, where it bound to CaMBD3 with an average of 4.5-fold higher affinity than to CaMBD2.

### Substitution of Trp-3587, but not Phe-3603, with Ala impairs CaM binding to CaMBD2

**Figure 7.**
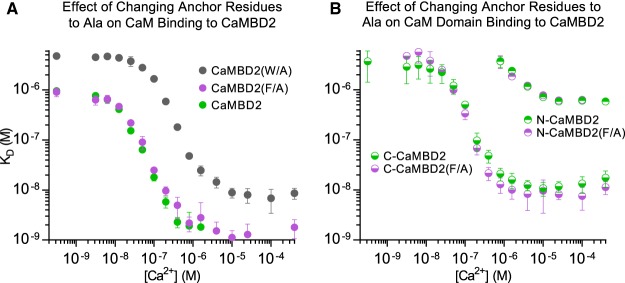
Effects of changing CaMBD2 Trp3587 and Phe3603 anchor residues to Ala on CaM binding. Effects of mutating RyR2 CaMBD2 anchor residues on the Ca^2+^-dependent affinities of (**A**) full-length CaM and (**B**) N- and C-domains for binding to CaMBD2. The binding model-fitted affinities (*K*_D_) are plotted as a function of [Ca^2+^]. Note the double logarithmic axes. Error bars represent the 95% confidence intervals of two replicates.

In the crystal structure of the CaM/RyR1CaMBD2 complex, the RyR2-equivalent Trp-3587 is an anchor site for the CaM C-domain, and RyR2 Phe-3603 is an anchor site for the CaM N-domain. We compared CaM titrations in the presence of the CaMBD2 peptide with titrations using peptides with either a Trp-3587-Ala (CaMBD2(W/A)) or a Phe-3603-Ala (CaMBD2(F/A)) substitution (Figure 7 and Supplementary Tables S1 and S2). The Ala substitution of Trp-3587 in CaMBD2 had a marked effect on CaM binding. Full-length CaM exhibited an average 30-fold lower affinity for CaMBD2(W/A), compared with CaMBD2 across the entire range of [Ca^2+^]. Moreover, the binding affinity was too weak to be measured when titrating CaMBD2(W/A) with the isolated CaM N- or C-domain (*K*_D_ > 5 µM, data not shown). This reduction in affinity of both CaM domains for CaMBD2(W/A) reveals that when isolated, both CaM domains prefer to bind around Trp-3587.

In contrast with the observations made for the CaMBD2(W/A) peptide, the Phe-3603-Ala substitution in CaMBD2 hardly affected the binding of CaM. There were no significant differences between the binding of full-length CaM or individual CaM domains to CaMBD2(F/A), compared with their binding to CaMBD2. However, at [Ca^2+^] > 1 µM, a trend towards slightly reduced or increased affinity of CaMBD2(F/A) was apparent for full-length CaM and the isolated C-domain, respectively (Figure 7). Hence, these results confirmed RyR2 Trp-3587 as a main CaM-binding determinant both at low and high [Ca^2+^], and suggested a much smaller contribution from Phe-3603 to the CaM–CaMBD2 interaction.

### Ca^2+^ affinities of CaM when complexed to each of the four RyR2 CaMBDs

**Figure 8.**
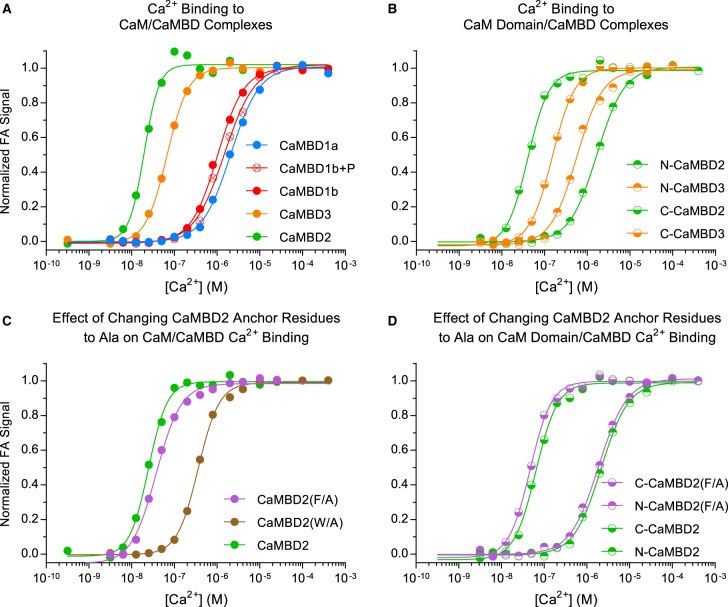
Visualization of Ca^2+^ binding to CaM/CaMBD complexes. Ca^2+^-binding curves (normalized FA signal as a function of [Ca^2+^]) are shown for full-length CaM/CaMBD complexes (**A**), for isolated CaM domain/CaMBD2 and -3 complexes (**B**), and for full-length CaM/CaMBD2 (**C**) or isolated CaM domains/CaMBD2 complexes (**D**) with CaMBD2 anchor residues changed to Ala. Solid lines show the fit of a generic Hill model for estimating CaM/CaMBD complex affinities for Ca^2+^ (see Supplementary Table S3).

We and others have previously demonstrated that CaM binds Ca^2+^ with higher affinity when bound to CaMBD2, compared with free CaM [10,45]. The experimental set-up used in this study allowed us to extend the analysis and estimate the CaM affinity for Ca^2+^ in the presence of each of the four CaMBDs. FA curves for the titration of protein–peptide complexes with [Ca^2+^] at CaM to peptide ratios >1 (excess of CaM) were extracted from the data and fitted to an empirical Hill model for Ca^2+^ binding to CaM (Figure 8 and Supplementary Table S3) (see ‘Experimental Procedures’ for details).

The CaM affinity for Ca^2+^ when in complex with each CaMBD was expressed as the apparent [Ca^2+^] required to saturate half of the CaM Ca^2+^- binding sites (app*K*_D_). CaM showed distinct affinities for binding Ca^2+^ when bound to either CaMBD1a, -1b, -1b + P, -2, or -3 as evident from the Ca^2+^ titration curves and corresponding app*K*_D_ values (Figure 8A and Supplementary Table S3). CaM displayed a 3.4-fold higher affinity for Ca^2+^ when complexed with CaMBD2 compared with CaMBD3 (app*K*_D_ 20 vs. 67 nM) and a markedly lower Ca^2+^ affinity when complexed with CaMBD1a, -1b, or -1b + P (app*K*_D_ 2.1, 1.0, and 1.5 µM, respectively).

The app*K*_D_ values were also fitted for the individual CaM domains bound to CaMBD2 or -3 for comparison with those of full-length CaM (Figure 8B and Supplementary Table S3). The CaM C-domain/CaMBD2 complex had a ∼2-fold lower affinity for Ca^2+^ compared with full-length CaM/CaMBD2 (app*K*_D_ 20 vs. 36 nM). On the other hand, the CaM N-domain/CaMBD2 complex had a 60-fold lower affinity for Ca^2+^ than full-length CaM/CaMBD2 (app*K*_D_ 20 nM vs. 1.2 µM). Notably, the affinity of the isolated CaM N-domain for Ca^2+^ when bound to CaMBD2 most likely reflects binding around Trp-3587 and not its putative binding site around Phe-3603 [7,35]. Similar to the observation for CaMBD2, the CaM C-domain/CaMBD3 complex showed a ∼2-fold lower affinity for Ca^2+^ compared with full-length CaM/CaMBD3 (app*K*_D_ 67 vs. 130 nM) (Figure 8B and Supplementary Table S3). Moreover, the CaM N-domain/CaMBD3 complex had a 6.7-fold lower affinity for Ca^2+^ than full-length CaM/CaMBD3 (app*K*_D_ 67 nM vs. 0.45 µM).

Investigation on the effect of changing the hydrophobic anchor sites of CaMBD2 revealed that the Trp-3587–Ala substitution markedly impaired Ca^2+^ binding to the CaM/CaMBD2(W/A) complex as the app*K*_D_ was ∼14-fold lower than for CaM/CaMBD2 (*K*_D_ 0.36 µM vs. 25 nM) (Figure 8C and Supplementary Table S3). The affinity of both CaM domains for CaMBD2(W/A) was too low to determine the Ca^2+^ affinity of the complexes. In contrast with CaM/CaMBD2(W/A), there was only a minor decrease in the Ca^2+^ affinity of the CaM/CaMBD2(F/A) complex compared with CaM/CaMBD2 (*K*_D_ 38 vs. 25 nM). Curiously, a minor increase in the Ca^2+^ affinity of the CaM C-domain (*K*_D_ 41 vs. 54 nM) was apparent when substituting Phe-3603 to Ala, whereas no difference was observed for the CaM N-domain/CaMBD2(F/A) complex (Figure 8D).

## Discussion

In this study, we used a two-dimensional titration assay in which the concentration of CaM was varied across columns and the Ca^2+^ concentration was varied across rows in a microtiter plate set-up (Figure 3) [37]. The row-wise titration with CaM was used to measure the CaM affinity for binding to RyR2 CaMBD peptides at 16 different Ca^2+^ concentrations. Experiments comparing full-length CaM with isolated CaM domains clearly showed that the CaM C-domain had a higher affinity for binding to CaMBD2 and -3, and therefore partially masked the contribution from the N-domain in full-length CaM (Figures 5 and 6, and Supplementary Tables S1 and S2). However, the CaM N-domain did contribute to the interaction with CaMBD2 and -3 as evident from full-length CaM having a higher affinity for either peptide than the isolated CaM C-domain (Figures 5 and 6, and Supplementary Tables S1 and S2).

**Figure 9.**
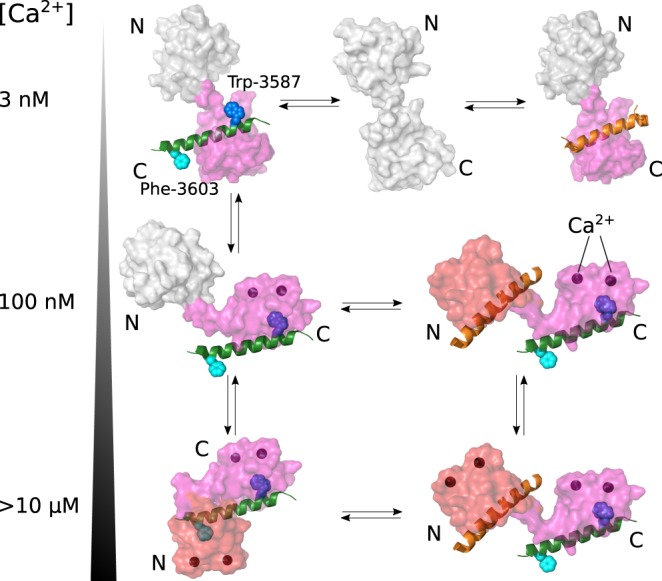
Proposed binding scheme for CaM regulation of RyR2 with increasing levels of cytosolic Ca^2+^. RyR2 CaMBD2 is indicated with a green α-helix and Ca^2+^ with dark spheres. When binding to RyR2, the CaM N-domain is highlighted in red and the C-domain in purple. Free domains are shown in white. At essentially Ca^2+^-free conditions (3 nM Ca^2+^, upper row), the CaM C-domain may bind to CaMBD2, involving Trp-3587, or to another RyR2 CaMBD (orange α-helix), such as CaMBD3. However, at resting cytosolic Ca^2+^ levels (∼100 nM, middle row), a Ca^2+^-saturated CaM C-domain binds to the CaMBD2 anchor residue, Trp-3587, and the apo-N-domain may interact with another CaMBD or may remain free. At cytosolic Ca^2+^ levels ∼1 µM and higher (illustrated as >10 µM, lower row), the CaM C-domain remains anchored to CaMBD2 Trp-3587, whereas the N-domain now binds Ca^2+^ and interacts with another site on RyR2 (e.g. CaMBD3), or clamps down on CaMBD2 Phe-3603.

Of the four RyR2 CaMBDs investigated, CaM had high affinities for CaMBD2 and CaMBD3, that increased with increasing [Ca^2+^]. Consistent with our results, Lau et al. [28] previously demonstrated a large increase in the CaM affinity for both CaMBD2 and -3 when comparing apo- and Ca^2+^-saturating conditions. Here, we demonstrate that CaM bound to CaMBD2 with higher affinity than to CaMBD3 in the range of [Ca^2+^] where we could reliably fit the data (13–200 nM [Ca^2+^]). Titrations with individual CaM domains revealed that this preference was mediated by the C-domain (Figure 6), which displayed higher affinity for CaMBD2 over CaMBD3 at all Ca^2+^ concentrations higher than 25 nM. This implies that the C-domain preferentially binds to CaMBD2 and thus leaves CaMBD3 available for a potential N-domain interaction (Figure 9). A potential CaM-mediated bridging of CaMBD2 and CaMBD3 was further supported by the higher affinity of the isolated CaM N-domain for binding to CaMBD3 compared with CaMBD2 at all Ca^2+^ concentrations tested. Lau et al. [28] made the same observations under Ca^2+^-free and -saturating conditions.

CaMBD2 is known to be pivotal for CaM-dependent inhibition of RyR channels and the RyR2 CaMBD2 Trp-3587 residue has been identified as the instrumental hydrophobic anchor for the binding of the CaM C-domain [6,8,10,15,28,32]. Similarly, Phe-3603 within RyR2 CaMBD2 was shown to be the hydrophobic anchor for the CaM N-domain in the crystal structure of the CaM/RyR1CaMBD2 complex [35]. In our experiments, the Trp-3587–Ala substitution prominently decreased the CaM affinity for CaMBD2 for both full-length CaM and the CaM C-domain across all [Ca^2+^] tested. This supports a key role of the Trp-3587 residue for the CaMBD2 interaction with the CaM C-domain in both the apo- and Ca^2+^-saturated state. Surprisingly, the Trp-3587 substitution to Ala also diminished the CaMBD2 affinity for the CaM N-domain, demonstrating that the CaM N-domain preferentially binds to the Trp-3587 site in the absence of the CaM C-domain. However, we do not anticipate this to be of physiological relevance, as the C-domain in the full-length CaM will outcompete the N-domain for binding to Trp-3587. An exception may be the presence of arrhythmogenic mutations where the C-domain Ca^2+^ binding is disrupted also in the presence of CaMBD2 [10].

Substitution of the proposed CaM N-domain anchor, Phe-3603-Ala, only marginally decreased the affinity of CaMBD2 for full-length CaM. Interestingly, this mutation marginally increased the affinity for the isolated CaM C-domain at [Ca^2+^] > 1 µM. These results suggest a potential contribution of RyR2 Phe-3603 to the CaM C-domain/CaMBD2 interaction at some, likely elevated, [Ca^2+^]. The Phe-3603 substitution to Ala caused no change in the CaM N-domain affinity for CaMBD2. This supported that the CaM N-domain prefers to bind around the Trp-3587 anchor site when not competing with the C-domain, and indicated that Phe-3603 is not a prerequisite for the CaM N-domain binding to RyR2 CaMBD2. The findings of this study were consistent with the ITC results presented by Lau et al. [28], concerning the reduced affinity of full-length CaM and its isolated C-domain for CaMBD2 when Trp-3587 was substituted with Ala. In general, our results indicate that data obtained from using individual CaM domains should be interpreted with caution. Given the structural and functional similarity between the CaM N- and C-domains, the isolated N-domain may preferentially bind to sites natively occupied by the C-domain (or *vice versa*) when otherwise using full-length CaM.

Compared with CaMBD2 and -3, CaM displayed an approximately three orders of magnitude lower affinity for binding to CaMBD1a and -1b. Thus, CaM would appear to bind preferentially to CaMBD2 or -3 by a wide margin compared with CaMBD1a and -1b, and under any [Ca^2+^] condition. However, since these CaMBDs are located in close proximity to the CaM-binding groove of RyR (Figure 2), their effective concentration relative to a proximate CaM may be very high. Accordingly, these two low-affinity CaMBDs may serve as secondary ‘bridging’ CaM-binding sites under some Ca^2+^ conditions or in certain RyR conformations, together with a high-affinity tethering site like that of CaMBD2. Noteworthy, Ser-2031 within RyR2 CaMBD1b is a target for phosphorylation by β-adrenergic-stimulated protein kinase A (PKA) [46,47] (Figure 1 and Table 1). Our results demonstrated a small decrease in the CaM affinity for the CaMBD1b peptide when using a Ser-2031 phosphorylated, compared with nonphosphorylated CaMBD1b. Hence, PKA-mediated Ser-2031 phosphorylation could potentially affect CaM's inhibition of RyR2, if CaMBD1b is indeed a functionally relevant RyR2 CaMBD at increased [Ca^2+^].

Aside from measuring the Ca^2+^-dependent propensity of CaM binding to the individual RyR2 CaMBD peptides, we also determined the CaM affinities for Ca^2+^ in the presence of the individual CaMBDs (expressed as an apparent *K*_D_, app*K*_D_) (Supplementary Table S3 and Figure 8A–D). In the presence of CaMBD2, full-length CaM had an app*K*_D_ of 20 nM, and the individual CaM C-domain had a similar app*K*_D_ of 36 nM (Figure 8A,B). This is in full agreement with our previous findings, measuring domain-wise intrinsic fluorescence (app*K*_D_ of 30 nM for the CaM C-domain in the presence of CaMBD2) [10,21]. Since the cytosolic [Ca^2+^] is maintained above 100 nM in the cardiomyocyte even at resting conditions, this strongly suggests that the C-domain of CaM is bound to CaMBD2 in a Ca^2+^-saturated state throughout the excitation contraction cycle. The Ca^2+^ affinity of the CaM N-domain in the presence of CaMBD2, however, was much lower (app*K*_D_ 1.2 µM). Although this affinity reflects the CaM N-domain bound around Trp-3587, and not Phe-3603 (see above), it is highly comparable to the Ca^2+^ affinity previously measured for the CaM N-domain in the context of full-length CaM using intrinsic protein fluorescence (app*K*_D_ of 0.8 µM for the CaM N-domain in the presence of CaMBD2) [10,21]. In the presence of CaMBD3, full-length CaM displayed an app*K*_D_ of 67 nM, and the isolated CaM C-domain an app*K*_D_ of 130 nM, thus slightly lower Ca^2+^ affinities than in the presence of CaMBD2 (Figure 8A,B). Intriguingly, the isolated CaM N-domain displayed an app*K*_D_ of 0.45 µM in the presence of CaMBD3 and thus a higher apparent Ca^2+^ affinity than in the presence of CaMBD2 (Figure 8B).

Altogether, our results add to our previously proposed model for the CaM–RyR2 interaction [10,21]: the CaM C-domain is constitutively anchored to CaMBD2 around Trp-3587 in a Ca^2+^-saturated state, whereas the CaM N-domain functions as the primary resident Ca^2+^ sensor under physiological Ca^2+^ oscillations (Figure 9). During cardiac excitation, when [Ca^2+^] rises above ∼0.5 µM, the CaM N-domain may bind to CaMBD3 (Figure 9). This would bridge RyR2 CaMBD2 and -3, similar to the observations made for the Ca^2+^-dependent CaM bridging of noncontiguous regions of both the voltage-gated Na^+^ (Na_V_1.5) and Ca^2+^ channels (Ca_V_1.2) [48–50]. Alternatively, if RyR2 CaMBD3 is not available for a CaM N-domain interaction, it is conceivable that at higher [Ca^2+^], the CaM N-domain may bind to CaMBD1a or -1b or clamp down onto the CaMBD2 around Phe-3603, or even bind a yet unidentified RyR2 site outside these regions (Figure 9). Whether CaMBD1a, -1b, and -3 are functionally relevant RyR2 CaMBDs remain to be investigated, but this study provides general guidelines for under which Ca^2+^ conditions these CaMBDs could be relevant.

CaM regulation of RyR2 Ca^2+^ release is essential for maintaining cardiac contraction cycles, and a thorough understanding of the molecular details of this regulation is important for evaluating the potential arrhythmogenic effects of novel CaM mutations [7,31,51]. In our binding model, the Ca^2+^-saturated CaM C-domain is pivotal for RyR2 inhibition, even at diastolic Ca^2+^ levels, and its high affinity for binding to CaMBD2 and for binding Ca^2+^ is therefore crucial. Subsequent Ca^2+^ binding to the CaM N-domain at elevated systolic Ca^2+^ levels changes CaM's binding mode and enhances channel inhibition. This is in accordance with the previously reported CaM-dependent decrease in RyR2 activity, especially above ∼0.5 µM Ca^2+^, as determined by ryanodine-binding experiments [52]. Finally, the arrhythmogenic CaM N-domain mutation, CaM-N53I (numbering without the start-Met), strongly corroborates a key role for the CaM N-domain in conveying information about increases in [Ca^2+^] to RyR2 [35].

## Conclusions

In conclusion, the results presented in this work provide the first report on the Ca^2+^ dependency of CaM binding to the four proposed CaMBDs of RyR2. Our data support a domain-wise interaction between CaM and RyR2 that changes with increasing cytosolic Ca^2+^ concentrations. The CaM C-domain is constitutively bound to CaMBD2 in a Ca^2+^-saturated state, and the CaM N-domain functions as a dynamic Ca^2+^ sensor, in the physiological range of Ca^2+^ concentrations, that can bridge noncontiguous regions of RyR2, such as CaMBD1a, -1b, or -3, or clamp down onto the Phe-3603 of CaMBD2.

## Abbreviations

CaM: calmodulin
CaMBD: CaM-binding domain
Ca_V_: voltage-gated Ca^2+^ channel
CICR: Ca^2+^-induced Ca^2+^ release
EDTA: ethylenediaminetetraacetic acid
EGTA: ethylene glycol-bis(β-aminoethyl ether)-*N*,*N*,*N*′,*N*′-tetraacetic acid
FA: fluorescence anisotropy
HD: helical domain
ITC: isothermal calorimetry
MBP: maltose-binding protein
Na_V_: voltage-gated Na^+^ channel
NTA: nitrilotriacetic acid
RyR: ryanodine receptor
SR: sarcoplasmic reticulum
TAMRA: carboxytetramethylrhodamine
