# Supplementary material for: Ca2+-dependent calmodulin binding to cardiac ryanodine receptor (RyR2) calmodulin-binding domains
Source: Biochem J. 2019 Jan 18;476(2):193–209. doi: 10.1042/BCJ20180545 (PMC6340113; doi:10.1042/BCJ20180545)
Supplement: Supplementary Tables [file BCJ-476-193-s1.pdf]

Table S1: Fitted K_D-values for full length CaM binding to RyR2 CaMBD peptides. 'Upper' and 'Lower' indicate the 95 % confidence intervals for the fitted K_D. Omitted data indicates that the affinity was either too low or high for reliable fitting i.e. K_D values outside the range 5 µM – 1 nM. K_D values are given in µM.

	Full length CaM																							
	CaMBD1a			CaMBD1b			CaMBD1b+P			CaMBD2			CaMBD3			CaMBD2(+)			CaMBD2(+)-W/A			CaMBD2(+)-F/A		
**[Ca^{2+}] (µM)**	**K_D**	Upper	Lower	**K_D**	Upper	Lower	**K_D**	Upper	Lower	**K_D**	Upper	Lower	**K_D**	Upper	Lower	**K_D**	Upper	Lower	**K_D**	Upper	Lower	**K_D**	Upper	Lower
**0.000**										**0.39**	0.46	0.31	**0.53**	0.58	0.48	**0.95**	1.02	0.88	**4.8**	5.3	4.2	**0.90**	1.08	0.73
**0.003**										**0.32**	0.37	0.27	**0.54**	0.57	0.51	**0.77**	0.86	0.68	**4.5**	5.2	3.9	**0.63**	0.77	0.50
**0.006**										**0.26**	0.30	0.23	**0.45**	0.48	0.42	**0.63**	0.66	0.61	**4.6**	5.0	4.3	**0.66**	0.78	0.53
**0.013**										**0.19**	0.22	0.17	**0.46**	0.51	0.41	**0.41**	0.43	0.38	**4.4**	4.4	4.4	**0.47**	0.53	0.40
**0.025**										**0.073**	0.085	0.062	**0.27**	0.29	0.25	**0.15**	0.17	0.13	**3.7**	4.6	2.9	**0.22**	0.23	0.21
**0.050**										**0.025**	0.030	0.019	**0.134**	0.155	0.113	**0.063**	0.072	0.055	**2.8**	3.1	2.5	**0.090**	0.117	0.063
**0.100**										**0.004**	0.007	0.001	**0.035**	0.041	0.029	**0.018**	0.021	0.015	**1.7**	1.8	1.5	**0.025**	0.027	0.022
**0.200**				**1.9**	2.1	1.7	**4.7**	5.4	4.1	**0.001**	0.003	0.000	**0.007**	0.009	0.006	**0.006**	0.007	0.004	**0.587**	0.627	0.546	**0.010**	0.012	0.008
**0.398**	**4.2**	4.7	3.8	**0.59**	0.66	0.52	**1.6**	2.0	1.3				**0.001**	0.002	0.001	**0.002**	0.003	0.002	**0.181**	0.205	0.156	**0.005**	0.007	0.003
**0.794**	**1.6**	1.7	1.4	**0.17**	0.21	0.14	**0.43**	0.47	0.40							**0.002**	0.004	0.000	**0.048**	0.055	0.041	**0.002**	0.003	0.001
**1.58**	**0.25**	0.27	0.23	**0.066**	0.079	0.054	**0.19**	0.21	0.17							**0.002**	0.003	0.001	**0.025**	0.030	0.019	**0.003**	0.006	0.000
**3.98**	**0.15**	0.16	0.14	**0.031**	0.046	0.016	**0.13**	0.16	0.09										**0.014**	0.018	0.011	**0.002**	0.002	0.001
**10.0**	**0.090**	0.097	0.084	**0.017**	0.024	0.010	**0.050**	0.071	0.030										**0.009**	0.011	0.007	**0.001**	0.002	0.001
**25.1**	**0.058**	0.062	0.054	**0.012**	0.015	0.009	**0.037**	0.052	0.022										**0.008**	0.011	0.005	**0.001**	0.002	0.000
**100**	**0.072**	0.077	0.067	**0.011**	0.015	0.007	**0.033**	0.039	0.027										**0.007**	0.010	0.003	**0.001**	0.002	0.000
**398**	**0.070**	0.075	0.066	**0.014**	0.017	0.010	**0.032**	0.037	0.026										**0.009**	0.011	0.007	**0.002**	0.003	0.001

Table S2: Fitted K_D-values for isolated CaM domains binding to RyR2 CaMBD peptides. Data entered as described for Table S1.

	**N-domain**												**C-domain**											
	CaMBD2			CaMBD2(+)			CaMBD2(+)-F/A			CaMBD3			CaMBD2			CaMBD2(+)			CaMBD2(+)-F/A			CaMBD3		
**[Ca^{2+}] (μM)**	**K_D**	Upper	Lower	K_D	Upper	Lower	**K_D**	Upper	Lower	**K_D**	Upper	Lower	**K_D**	Upper	Lower	K_D	Upper	Lower	**K_D**	Upper	Lower	**K_D**	Upper	Lower
**0.000**													**5.7**	6.3	5.1	**3.7**	5.9	1.4	**9.0**	13.7	4.2	**4.0**	4.7	3.4
**0.003**													**4.2**	5.5	2.9	**2.9**	4.5	1.3	**4.7**	7.3	2.1	**3.3**	6.0	0.6
**0.006**													**4.6**	4.9	4.3	**3.1**	4.6	1.6	**5.7**	8.0	3.4	**4.4**	5.7	3.0
**0.013**													**3.7**	4.9	2.5	**2.6**	3.9	1.4	**3.8**	5.7	1.9	**3.0**	5.5	0.5
**0.025**													**1.7**	1.9	1.5	**2.2**	3.1	1.3	**2.5**	2.8	2.2	**3.5**	4.0	3.0
**0.050**													**0.76**	0.96	0.56	**1.2**	1.6	0.8	**1.0**	1.1	0.9	**2.1**	3.3	1.0
**0.100**													**0.22**	0.27	0.17	**0.50**	0.54	0.47	**0.34**	0.42	0.25	**1.5**	1.6	1.3
**0.200**													**0.044**	0.057	0.030	**0.097**	0.136	0.059	**0.068**	0.086	0.050	**0.49**	0.53	0.45
**0.398**	**4.2**	7.7	0.6							**2.0**	2.7	1.2	**0.013**	0.017	0.009	**0.048**	0.062	0.034	**0.022**	0.028	0.015	**0.19**	0.21	0.16
**0.794**	**2.1**	2.7	1.4	**3.7**	4.6	2.8	**3.7**	4.8	2.6	**0.64**	0.72	0.55	**0.008**	0.010	0.005	**0.021**	0.027	0.015	**0.013**	0.017	0.008	**0.071**	0.090	0.051
**1.58**	**1.5**	1.7	1.3	**2.4**	2.8	2.0	**1.9**	2.1	1.6	**0.26**	0.31	0.22	**0.005**	0.007	0.003	**0.016**	0.021	0.011	**0.010**	0.014	0.007	**0.050**	0.064	0.037
**3.98**	**0.95**	1.01	0.89	**1.2**	1.2	1.1	**1.2**	1.3	1.1	**0.14**	0.18	0.10	**0.005**	0.007	0.003	**0.012**	0.015	0.010	**0.008**	0.013	0.004	**0.040**	0.050	0.029
**10.0**	**0.53**	0.60	0.46	**0.71**	0.76	0.67	**0.78**	0.83	0.73	**0.088**	0.102	0.075	**0.004**	0.006	0.003	**0.011**	0.014	0.007	**0.010**	0.016	0.003	**0.034**	0.044	0.025
**25.1**	**0.34**	0.37	0.32	**0.58**	0.63	0.54	**0.62**	0.66	0.57	**0.075**	0.096	0.054	**0.004**	0.006	0.002	**0.012**	0.015	0.009	**0.008**	0.010	0.006	**0.032**	0.040	0.024
**100**	**0.36**	0.45	0.28	**0.61**	0.66	0.55	**0.63**	0.67	0.59	**0.085**	0.110	0.059	**0.004**	0.007	0.002	**0.013**	0.018	0.008	**0.008**	0.011	0.004	**0.036**	0.051	0.021
**398**	**0.32**	0.42	0.21	**0.59**	0.63	0.54	**0.59**	0.65	0.53	**0.095**	0.104	0.087	**0.006**	0.008	0.003	**0.017**	0.024	0.011	**0.011**	0.015	0.008	**0.039**	0.053	0.025

Table S3: Apparent Ca^{2+} affinities of CaM/CaMBD complexes. Affinities are expressed as the [Ca^{2+}] required for saturating half of the protein-peptide complex with Ca^{2+} ($appK_D$). 'Upper' and 'Lower' indicate the 95 % confidence interval for the fitted $appK_D$.

	**$appK_D$ (μM)**	**Upper**	**Lower**	**n**	**Upper**	**Lower**
CaM/CaMBD1a	**2.1**	2.3	1.8	**1.3**	1.5	1.1
CaM/CaMBD1b	**1.0**	1.1	0.9	**1.4**	1.5	1.3
CaM/CaMBD1b+P	**1.5**	1.7	1.3	**1.2**	1.4	1.0
CaM/CaMBD2	**0.020**	0.021	0.018	**2.5**	3.0	1.9
CaM/CaMBD3	**0.067**	0.072	0.063	**1.7**	1.9	1.6
CaM/CaMBD2(+)	**0.025**	0.026	0.023	**2.0**	2.2	1.7
CaM/CaMBD2(+)-W/A	**0.36**	0.38	0.34	**1.8**	1.9	1.6
CaM/CaMBD2(+)-F/A	**0.038**	0.042	0.033	**1.5**	1.7	1.3
N-domain/CaMBD2	**1.2**	1.3	1.2	**1.4**	1.5	1.3
N-domain/CaMBD3	**0.45**	0.49	0.41	**1.5**	1.7	1.3
N-domain/CaMBD2(+)	**1.5**	1.7	1.4	**1.3**	1.4	1.2
N-domain/CaMBD2(+)-F/A	**1.5**	1.6	1.4	**1.4**	1.5	1.3
C-domain/CaMBD2	**0.036**	0.040	0.032	**1.9**	2.2	1.5
C-domain/CaMBD3	**0.13**	0.14	0.11	**1.6**	1.8	1.4
C-domain/CaMBD2(+)	**0.054**	0.061	0.048	**1.7**	1.9	1.4
C-domain/CaMBD2(+)-F/A	**0.041**	0.046	0.036	**1.7**	2.1	1.4
